# Efficacy of Postoperative Adjuvant Transcatheter Arterial Chemoembolization in Hepatocellular Carcinoma Patients With Microscopic Portal Vein Invasion

**DOI:** 10.3389/fonc.2022.831614

**Published:** 2022-06-20

**Authors:** Yiwen Qiu, Yi Yang, Tao Wang, Shu Shen, Wentao Wang

**Affiliations:** Department of Liver Surgery & Liver Transplantation Center, West China Hospital of Sichuan University, Chengdu, China

**Keywords:** Hepatocellular carcinoma, microvascular invasion, microscopic portal vein invasion, prognostic factor, adjuvant treatment

## Abstract

**Background:**

Microscopic portal vein invasion (MPVI) strongly predicts poor prognosis in patients with hepatocellular carcinoma (HCC). This study aims to investigate the impact of MPVI on the efficacy of postoperative adjuvant transcatheter arterial chemoembolization (PA-TACE).

**Methods:**

From April 2014 to July 2019, a total of 512 HCC patients who underwent curative liver resection (LR) with microscopic vascular invasion (MVI) confirmed by histopathological examination were enrolled and divided into LR alone and PA-TACE groups. They were subsequently stratified into subgroups according to the presence of MPVI. Recurrence-free survival (RFS) and overall survival (OS) were compared using Kaplan–Meier curves and the log-rank test. The efficacy of PA-TACE was tested using univariate and multivariate Cox regression analyses. Sensitivity analysis was conducted after propensity score matching (PSM).

**Results:**

Among all patients, 165 (32.3%) patients underwent PA-TACE, and 196 (38.2%) patients presented MPVI. In the entire cohort, PA-TACE and the presence of MPVI were identified as independent predictors for RFS and OS (all p<0.05). In the subgroup analysis, patients without MPVI who received PA-TACE had significantly better outcomes than those who underwent LR alone before and after PSM (all p<0.05). For patients with MPVI, PA-TACE displayed no significant benefit in terms of improving either RFS or OS, which was consistent with the results from the PSM cohort.

**Conclusion:**

Among the HCC patients without MPVI who underwent curative liver resection, those who received PA-TACE had better RFS and OS outcomes than those who underwent LR alone. For patients with MPVI, PA-TACE had no significant effect on either RFS or OS outcomes.

## Introduction

Primary liver cancer is the sixth most commonly diagnosed cancer and the third leading cause of cancer-related death worldwide, ranking fifth in terms of global incidence and second in terms of mortality for men (1). The most prevalent type of primary liver cancer is hepatocellular carcinoma (HCC), which comprises 75%-85% of all cases (2). In most regions with a high incidence of HCC, represented by eastern China, one of the key determinants is chronic HBV infection (3). The optimal therapeutics for early-stage HCC remains to be curative liver resection (LR) and liver transplantation in most instances (4). Unfortunately, it has a 5-year recurrence rate of over 50%, significantly increasing tumor-related mortality (5, 6). Thus, adjuvant therapies such as postoperative adjuvant transcatheter arterial chemoembolization (PA-TACE), kinase inhibitors and immune checkpoint inhibitors have been explored in many trials and are now utilized to improve the outcomes of liver resection (7, 8).

It is important to discover strong predictors of postoperative outcomes so that the proper population can be selected for adjuvant therapy to prevent recurrence (9). Microscopic vascular invasion (MVI), characterized as cancer cells invading the microscopic vessels in the peritumor liver parenchyma, has been perceived as a strong predictor for tumor recurrence and unsatisfactory overall survival (OS) among patients with HCC postoperatively (10). However, due to the various definitions and descriptions of MVI in different studies, the actual effect of MVI on the prognosis of HCC patients, particularly during the early stage, remains unclear and debated (11). A recent study suggested that MVI should be additionally segregated into microscopic vessel invasion (MI, defined as newly developed microscopic vascular structures in the peritumor liver parenchyma; microvessels have no obvious features of portal or hepatic vein or hepatic artery) and microscopic portal vein invasion (MPVI). The subclassification of MVI significantly affects the prognosis of HCC after liver resection (12) and can guide the selection of appropriate recipients for PA-TACE.

A variety of strategies employing PA-TACE have been proposed over the years, aiming to reduce recurrence after curative hepatectomy. Generally, PA-TACE is recommended for HCC patients at high recurrent risk (e.g., those have MVI, multiple lesions or gross vascular invasion) (13–15). An overall oncologic benefit of PA-TACE has been observed in selected patients (16). However, multiple studies have provided various and often conflicting results on the potency of PA-TACE in different patient populations (17). Regarding post-hepatectomy patients with MVI, the benefits of PA-TACE remain controversial (18). Therefore, it is necessary to explore which patients receive benefits from PA-TACE based on MVI subclassification.

In the present study, we explored the benefit of PA-TACE based on MVI subclassification. The outcomes of PA-TACE were evaluated in a large cohort of HCC patients who underwent curative LR and were stratified by the presence of MPVI using propensity score matching (PSM).

## Methods

### Patients

**Figure 1:**
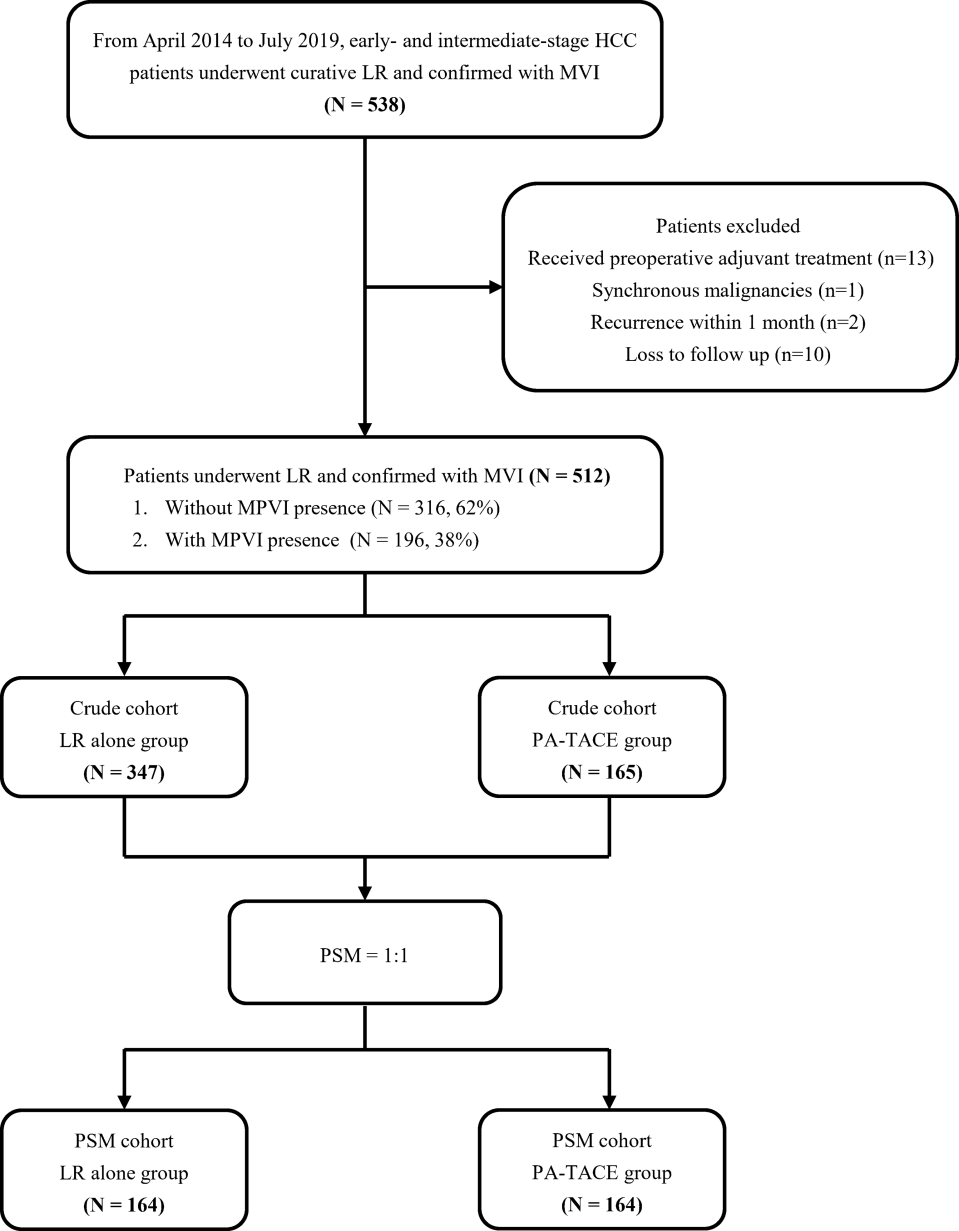
Diagram of patient selection.

This study was approved by the Ethics Committee of West China Hospital of Sichuan University (No. 2019-788) and was conducted in accordance with the Declaration of Helsinki. From April 2014 to July 2019, in West China Hospital, we studied the patient population with the following features in the present retrospective study. The inclusion criteria including 1) patients who underwent R0 liver resection confirmed at histopathological examination for early- or intermediate-stage HCC; 2) HCC confirmed by postoperative histopathological examination with MVI (i.e. microscopic tumor cells detected in the portal or hepatic vein of the surrounding liver parenchyma) (19); and 3) TACE as the only adjuvant treatment. The following were the exclusion criteria: 1) patients who received additional treatment (such as TACE, radiotherapy and sorafenib treatment) prior to curative LR; 2) with other synchronous malignancies or remote or lymph node metastasis; 3) with cardiopulmonary, renal, or cerebral dysfunction before LR; 4) with early recurrence within 1 month; and 5) loss to follow-up within 2 months after discharge. The patient selection flowchart is displayed in **Figure 1**.

### Treatment

Following necessary preoperative examination, all patients initially underwent curative LR. postoperative histopathological examination confirmed a negative resection margin, and no detectable tumors were found by imaging examination in the remanent liver. Patient baseline data and other intraoperative parameters were collected. We recommended all patients to received PA-TACE at 1-3 months after the operation when their liver function reached the standard: Child-Pugh class A/B, with a serum bilirubin level 1.5 times the normal upper limit, alanine aminotransferase (ALT) and aspartate aminotransferase (AST) levels two times the normal upper limit. During PA-TACE, liver angiography and CT angiography was conducted to reveal any possible recurrence in the residual liver. Simultaneously, if no recurrence was found, an arterial catheter was inserted through the femoral artery using the Seldinger technique. We inserted the catheter into right or left hepatic artery. Through the catheter, we injected chemotherapeutic agents doxorubicin (20~30 mg/m^2^) or pharmorubicin (20~40 mg/m^2^) and lipiodol (3~5 mL). The dosage of lipiodol and doxorubicin was determined by body surface area and underlying liver function.

### Subclassification of MVI

MVI was subclassified as follows (12): 1) microvessel invasion (MI), 2) microscopic portal vein invasion (MPVI), 3) microscopic hepatic vein invasion (MHVI), or 4) microscopic hepatic artery invasion (MHAI). Microvessels were prescribed as microscopic vascular structures resulting from angiogenesis in the tumor capsule or peritumor normal liver parenchyma; these microvessels have no obvious features of portal or hepatic vein or hepatic artery. In this study, MVI was subcategorized into a) without MPVI and b) with MPVI. Moreover, other pathological manifestations, including differentiation of tumor cells, microsatellites, and cirrhosis of liver parenchyma, were also recorded.

### Follow-Up

In the first 2 years, the patients returned for follow-up visit every 2 months and received liver function assessments, tumor markers, and abdominal ultrasounds every time. All patients were followed up once every 3 months from the third year onward. We performed contrast-enhanced computed tomography (CT) or magnetic resonance imaging (MRI) at an 6-months interval or when recurrence/metastasis was suspected. Patients with CT or MRI images showing rapid tumor staining in the arterial phase that disappeared in the early venous phase combined with an increase in the serum AFP level were determined as recurrence. The main endpoint of the study was overall survival (OS, defined as the period between discharge and death), while recurrence-free survival (RFS, calculated from the date of surgery to the date when recurrence and/or metastasis was confirmed) was the secondary endpoint.

### Statistical Analysis

Parametric continuous data are expressed as the mean ± standard deviation (SD), and nonparametric data are expressed as the medians and interquartile ranges (Q1–Q3). Categorical data are expressed as numbers and percentages. Continuous variables were compared by Student’s t test or the Mann–Whitney U test. Categorical variables were evaluated using the χ2 test or Fisher’s exact test, as appropriate. Kaplan–Meier curves and the log-rank test were used to analyze DFS and OS. PA-TACE efficacy based on the MVI subclassification subgroups was assessed by Cox proportional hazards regression models. Propensity score matching (PSM) was used to reduce the differences in baseline characteristics. Variables that were not balanced in the LR alone group and PA-TACE group were entered into the propensity model with the use of a 1:1 matching protocol without replacement, with a caliper width equal to 0.02 of the SD of the logit of the propensity score. Kaplan–Meier curves and Cox regression analysis were conducted as sensitivity analyses for both the crude and PSM cohorts.

A 2-tailed P value<0.05 was considered to be statistically significant in all analyses. Empower (R) (www.empowerstats.com; X&Y solutions, Inc., Boston MA) was used for all statistical analyses.

## Results

### Baseline and Clinicopathological Data of Patients

We enrolled 512 patients in this study, with 165 patients received PA-TACE. No serious adverse reactions occured. As displayed in **Table 1**, the BMI of patients who underwent LR alone was significantly lower than that of the PA-TACE group (22.5 ± 3.0 vs. 23.4 ± 2.9, p=0.010). Regarding perioperative data, the patients in the LR group had more blood loss (300 (10–7000) ml vs. 300 (15–5500) ml, p=0.003), more frequently required blood transfusion (10.4% vs. 3.6%, p=0.009) and more frequently had MPVI (42.4% vs. 29.7%, p=0.006) than patients in the PA-TACE group (**Table 2**). PSM process selected 164 pairs of patients who did and did not received PA-TACE with no significantly different variables between the two groups. The median follow-up time was 26 (3-60) months for the crude cohort and 27 (3-60) months for the PSM cohort. The median interval between surgery and PA-TACE was 42 (28-97) days.

**Table 1:** Baseline data of all patients stratified by treatment.

Variables	Crude cohort			PSM cohort		
	LR (n = 347)	PA-TACE (n = 165)	P value	LR (n = 164)	PA-TACE (n = 164)	P value
**Age, y**	52 ± 11	50 ± 12	0.565	52 ± 12	51 ± 12	0.214
**BMI, kg/m^2^**	22.6 ± 3.0	23.4 ± 2.9	0.010	22.8 ± 2.8	23.3 ± 2.8	0.137
**Diameter, cm**	4.9 ± 2.9	4.8 ± 2.9	0.531	5.0 ± 2.9	4.7 ± 2.9	0.278
**PLT, ×10^9^/L**	161.8 ± 81.7	159.6 ± 83.3	0.649	145.0 (31.0-454.0)	138.0 (42.0-610.0)	0.807
**ALT, U/L**	49.7 ± 44.6	49.7 ± 57.2	0.598	37.0 (11.0-342.0)	35.0 (10.0-629.0)	0.991
**TBIL, μmol/L**	15.5 ± 16.1	15.9 ± 9.8	0.424	14.5 (2.4-44.6)	13.6 (3.5-100.0)	0.859
**ALB, g/L**	42.0 ± 4.4	41.9 ± 3.6	0.756	41.8 (27.1-52.4)	41.9 (28.9-55.3)	0.977
**INR**	1.1 ± 0.1	1.1 ± 0.1	0.991	1.0 (0.8-1.4)	1.0 (0.9-1.4)	0.913
**Sex**			0.255			0.261
**male**	305 (87.9%)	139 (84.2%)		145 (88.4%)	138 (84.1%)	
**female**	42 (12.1%)	26 (15.8%)		19 (11.6%)	26 (15.9%)	
**Alcohol consumption**			0.568			0.310
**no**	209 (60.2%)	95 (57.6%)		103 (62.8%)	94 (57.3%)	
**yes**	138 (39.8%)	70 (42.4%)		61 (37.2%)	70 (42.7%)	
**Hypertension**			0.422			0.592
**no**	305 (87.9%)	149 (90.3%)		145 (88.4%)	148 (90.2%)	
**yes**	42 (12.1%)	16 (9.7%)		19 (11.6%)	16 (9.8%)	
**Diabetes**			0.67			0.792
**no**	327 (94.2%)	157 (95.2%)		157 (95.7%)	156 (95.1%)	
**yes**	20 (5.8%)	8 (4.8%)		7 (4.3%)	8 (4.9%)	
**HBsAg**			0.683			0.884
**negative**	56 (16.1%)	29 (17.6%)		28 (17.1%)	29 (17.7%)	
**positive**	291 (83.9%)	136 (82.4%)		136 (82.9%)	135 (82.3%)	
**AFP, ng/mL**			0.054			0.054
**≥400**	208 (59.9%)	84 (50.9%)		102 (62.2%)	84 (51.2%)	
**<400**	139 (40.1%)	81 (49.1%)		62 (37.8%)	80 (48.8%)	
**Number of tumors**			0.092			0.273
**single**	231 (66.6%)	122 (73.9%)		112 (68.3%)	121 (73.8%)	
**multiple**	116 (33.4%)	43 (26.1%)		52 (31.7%)	43 (26.2%)	
**Child–Pugh classification**			0.835			1.000
**A**	342 (98.6%)	163 (98.8%)		162 (98.8%)	162 (98.8%)	
**B**	5 (1.4%)	2 (1.2%)		2 (1.2%)	2 (1.2%)	

PSM, propensity score matching; PA-TACE, postoperative adjuvant transhepatic arterial chemoembolization; TBIL, total bilirubin; TP, total protein; ALB, albumin; ALT, alanine aminotransferase; AFP, alpha-fetoprotein; PLT, platelets; INR, international normalized ratio; HBsAg, hepatitis B surface antigen.

**Table 2:** Surgical and pathological data of all patients stratified by treatment.

Variables	Crude cohort			PSM cohort		
	LR (n=347)	PA-TACE (n = 165)	P value	LR (n = 165)	PA-TACE (n = 165)	P value
**Blood loss, mL**	300 (10-7000)	300 (15-5500)	0.003	300 (10-6000)	275 (15-5500)	0.110
**Operation time, min**	230 (50-555)	240 (60-430)	0.984	228 (100-505)	240 (60-430)	0.897
**Approach of resection**			0.150			0.897
**Nonanatomical**	189 (54.5%)	101 (61.2%)		98 (59.8%)	100 (61.0%)	
**Anatomical**	158 (45.5%)	64 (38.8%)		66 (40.2%)	64 (39.0%)	
**Pringle maneuver**			0.693			0.741
**no**	44 (12.7%)	23 (13.9%)		20 (12.2%)	22 (13.4%)	
**yes**	303 (87.3%)	142 (86.1%)		144 (87.8%)	142 (86.6%)	
**Blood transfusion**			0.009			0.777
**no**	311 (89.6%)	159 (96.4%)		157 (95.7%)	158 (96.3%)	
**yes**	36 (10.4%)	6 (3.6%)		7 (4.3%)	6 (3.7%)	
**Differentiation**			0.670			0.910
**grades I-II**	132 (38.0%)	66 (40.0%)		66 (40.2%)	65 (39.6%)	
**grades III-IV**	215 (62.0%)	99 (60.0%)		98 (59.8%)	99 (60.4%)	
**Microsatellites**			0.195			1.000
**no**	285 (82.1%)	143 (86.7%)		142 (86.6%)	142 (86.6%)	
**yes**	62 (17.9%)	22 (13.3%)		22 (13.4%)	22 (13.4%)	
**MPVI**			0.006			0.476
**no**	200 (57.6%)	116 (70.3%)		109 (66.5%)	115 (70.1%)	
**yes**	147 (42.4%)	49 (29.7%)		55 (33.5%)	49 (29.9%)	
**Cirrhosis**			0.548			0.377
**no**	176 (50.7%)	79 (47.9%)		86 (52.4%)	78 (47.6%)	
**yes**	171 (49.3%)	86 (52.1%)		78 (47.6%)	86 (52.4%)	

PSM, propensity score matching; PA-TACE, postoperative adjuvant transhepatic arterial chemoembolization; MPVI, microportal vein invasion.

According to the postoperative histopathological examination, 42.4% (147/347) of the HCC patients who underwent LR alone presented MPVI versus 29.7% (49/165) of those who received PA-TACE. This difference in the incidence of MPVI was significant (p=0.006). The other pathological parameters showed no significant differences between the groups.

### Overall Effect of PA-TACE on the RFS and OS Outcomes of HCC Patients With MVI

**Figure 2:**
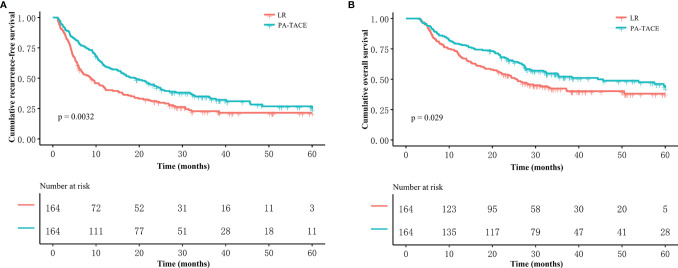
Kaplan–Meier analysis of RFS and OS stratified by treatment in the PSM cohort. Patients who received PA-TACE had better RFS **(A)** and OS **(B)** than those who underwent liver resection alone.

In the PSM cohort, the median RFS time for patients who underwent LR alone was shorter than that for patients who received PA-TACE (8.7 vs. 19.0 months, p=0.0032, **Figure 2A**). The median OS time of the HCC patients with MVI was 24.6 months for the LR alone group and 44.4 months for the PA-TACE group. The OS of PA-TACE group was significantly superior to that for the LR alone group (1-, 3-, 5-year rates, 79.3%, 52.1%, 42.9% vs. 71.3%, 42.3%, 38.0%, p=0.029) in the PSM cohort (**Figure 2B**).

Univariate and multivariate regression analyses (**Table 3**) suggested that PA-TACE (HR 0.720, 95% CI 0.554-0.935, p= 0.014) and the presence of MPVI (HR 1.543, 95% CI 1.175-2.027, p=0.002) were independent predictors for RFS. Regarding OS, PA-TACE (HR 0.744, 95% CI 0.551-0.973, p= 0.041) and the presence of MPVI (HR 1.675, 95% CI 1.235-2.272, p=0.001) were also identified as independent predictors. The correlated results of the crude cohort were reported in **Supplementary File**, **Figure S1** and **Table S1**.

**Table 3:** Univariate and multivariate analysis for RFS and OS in HCC patients with MVI in the PSM cohort.

Variables	RFS				OS			
	Univariate		Multivariate		Univariate		Multivariate	
	HR (95% CI)	p value	HR (95% CI)	p value	HR (95% CI)	p value	HR (95% CI)	p value
**SEX, male**	0.787 (0.536,1.156)	0.222			1.038 (0.685, 1.575)	0.859		
**Age, y**	**0.987 (0.976,0.999)**	**0.030**	**0.985 (0.974,0.997)**	**0.011**	0.991 (0.978, 1.004)	0.160		
**BMI, kg/m^2^**	1.013 (0.968,1.060)	0.567			1.007 (0.956, 1.061)	0.785		
**Alcohol consumption, yes**	1.216 (0.937,1.577)	0.141			1.062 (0.785, 1.436)	0.695		
**Hypertension, yes**	0.676 (0.427,1.069)	0.094			0.635 (0.361, 1.117)	0.115		
**Diabetes, yes**	**0.415 (0.185,0.935)**	**0.034**	0.529 (0.233,1.202)	0.128	0.511 (0.210, 1.243)	0.139		
**HBsAg, positive**	1.408 (0.986,2.010)	0.060			**1.654 (1.067, 2.564)**	**0.025**	**1.714 (1.104, 2.662)**	**0.016**
**AFP, <400 ng/mL**	**0.715 (0.550,0.929)**	**0.012**	**0.741 (0.566,0.970)**	**0.029**	**0.734 (0.542, 0.995)**	**0.046**	0.762 (0.562, 1.034)	0.081
**Diameter, cm**	1.000 (0.955,1.046)	0.987			1.006 (0.957, 1.058)	0.807		
**Number of tumors, multiple**	**1.783 (1.360,2.338)**	**<0.001**	**1.473 (1.110,1.953)**	**0.007**	**1.588 (1.164, 2.164)**	**0.003**	1.281 (0.923, 1.777)	0.138
**PLT, ×10^9^/L**	0.999 (0.998,1.001)	0.535			1.000 (0.998, 1.002)	0.769		
**ALT, U/L**	1.000 (0.998,1.003)	0.831			1.000 (0.997, 1.003)	0.558		
**TBIL, μmol/L**	1.013 (0.996,1.029)	0.129			1.011 (0.992, 1.030)	0.919		
**ALB, g/L**	1.000 (0.970,1.032)	0.985			0.990 (0.956, 1.025)	0.557		
**INR**	0.820 (0.195,3.445)	0.787			0.506 (0.095, 2.692)	0.424		
**Child–Pugh classification, B**	1.304 (0.484,3.510)	0.600			2.091 (0.775, 5.643)	0.145		
**Approach of resection, anatomical**	1.092 (0.841,1.418)	0.509			1.293 (0.961, 1.741)	0.090		
**Pringle maneuver, yes**	0.760 (0.530,1.090)	0.136			0.883 (0.582, 1.340)	0.559		
**Blood loss, mL**	1.000 (1.000,1.000)	0.375			1.000 (1.000, 1.000)	0.110		
**Blood transfusion, yes**	1.409 (0.768,2.582)	0.268			1.750 (0.924, 3.313)	0.086		
**Operation time, min**	1.001 (1.000,1.003)	0.086			1.001 (0.999, 1.003)	0.338		
**PA-TACE, yes**	**0.681 (0.527,0.881)**	**0.003**	**0.720 (0.554,0.935)**	**0.014**	**0.719 (0.534, 0.967)**	**0.029**	**0.744 (0.551, 0.973)**	**0.041**
**Differentiation, grades 3-4**	1.150 (0.883,1.497)	0.301			1.055 (0.780, 1.429)	0.727		
**Microsatellites, yes**	**1.782 (1.257,2.527)**	**0.001**	1.542 (1.070,2.222)	0.020	**1.814 (1.234, 2.667)**	**0.002**	**1.613 (1.077, 2.415)**	**0.020**
**MPVI, yes**	**1.621 (1.239,2.121)**	**<0.001**	**1.543 (1.175,2.027)**	**0.002**	**1.705 (1.261, 2.306)**	**0.001**	**1.675 (1.235, 2.272)**	**0.001**
**Cirrhosis, yes**	1.088 (0.842,1.406)	0.51772			0.970 (0.722, 1.302)	0.837		

RFS, recurrence free survival; OS, overall survival; HCC, hepatocellular carcinoma; PSM, propensity score matching; BMI, body mass index; HBsAg: hepatitis B surface antigen; AFP, alpha-fetoprotein; TBIL, total bilirubin; TP, total protein; ALB, albumin; ALT, alanine aminotransferase; PLT, platelets; INR, international normalized ratio; PA-TACE, postoperative adjuvant transhepatic arterial chemoembolization; MPVI, microportal vein invasion.

Bold values, statistical significant.

### Effect of PA-TACE on RFS and OS Outcomes Based on the Subclassification of MVI

**Figure 3:**
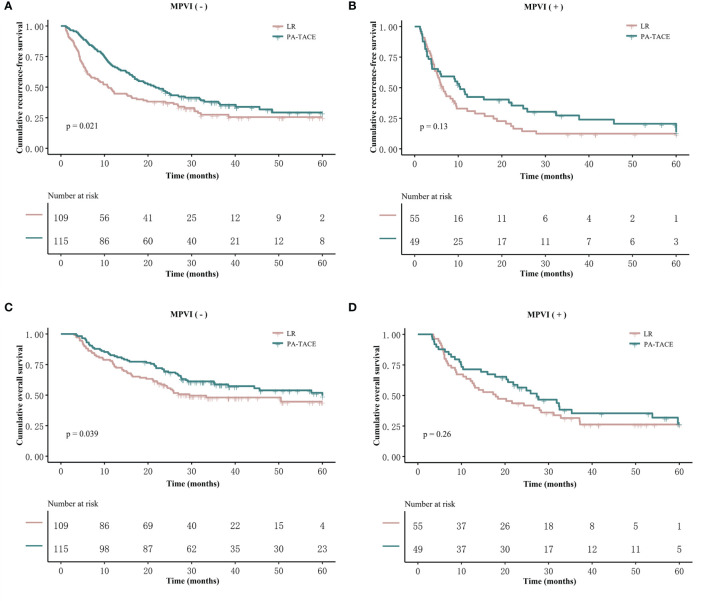
Kaplan–Meier analysis of RFS and OS stratified by treatment and the presence or absence of MPVI in the PSM cohort. Patients without MPVI who received PA-TACE had better RFS **(A)** and OS **(C)** than those who underwent liver resection alone. The RFS **(B)** and OS **(D)** of patients with MPVI showed no significant differences between the PA-TACE and LR alone groups.

In the PSM cohort, among patients without MPVI, the RFS of the PA-TACE group was significantly better than that of the LR group (median RFS, 21.7 vs. 10.9 months; 1-, 3-, and 5-year rates, 67%, 38.1%, and 29.3% vs. 46.5%, 27.4%, and 25.5%, p=0.021, **Figure 3A**). Similarly, the OS of the PA-TACE group was significantly better than that of the LR group in patients without MPVI (median OS, 57.5 vs. 29.5 months; 1-, 3-, 5-year rates, 82.6%, 58.8%, 49.6% vs. 75.2%, 48.1%, 44.6%, p=0.039, **Figure 3C**). Moreover, univariate and multivariate regression analysis (**Table 4**) suggested that PA-TACE was a significant protective factor for both RFS (HR 0.726, 95% CI 0.475-0.986, p=0.043) and OS (HR 0.691, 95% CI 0.500-0.955, p=0.025). The correlated results of the crude cohort were reported in **Supplementary File**, **Figure S2A, C** and **Table S2**.

**Table 4:** Univariate and multivariate analysis for RFS and OS in HCC patients without MPVI in the PSM cohort.

Variables	RFS				OS			
	Univariate		Multivariate		Univariate		Multivariate	
	HR (95% CI)	p value	HR (95% CI)	p value	HR (95% CI)	p value	HR (95% CI)	p value
**SEX, male**	1.218 (0.725, 2.045)	0.456			0.824 (0.509, 1.333)	0.431		
**Age, y**	**0.982 (0.966, 0.998)**	**0.031**	**0.980 (0.963, 0.997)**	**0.018**	**0.982 (0.968, 0.996)**	**0.012**	**0.980 (0.967, 0.994)**	**0.005**
**BMI, kg/m2**	1.007 (0.940, 1.079)	0.840			1.009 (0.952, 1.069)	0.766		
**Alcohol consumption, yes**	0.975 (0.659, 1.444)	0.900			1.322 (0.957, 1.827)	0.090		
**Hypertension, yes**	**0.449 (0.208, 0.966)**	**0.040**	0.889 (0.595, 1.329)	0.567	0.653 (0.383, 1.113)	0.117		
**Diabetes, yes**	0.434 (0.138, 1.369)	0.154			0.470 (0.192, 1.147)	0.097		
**HBsAg, positive**	**1.844 (1.011, 3.362)**	**0.046**	**2.192 (1.146, 4.190)**	**0.018**	1.410 (0.897, 2.217)	0.137		
**AFP, <400 ng/mL**	0.727 (0.491, 1.075)	0.110			1.000 (1.000, 1.000)	0.420		
**Diameter, cm**	0.956 (0.888, 1.029)	0.229			0.979 (0.921, 1.040)	0.494		
**Number of tumors, multiple**	**1.830 (1.224, 2.736)**	**0.003**	**1.563 (1.012, 2.414)**	**0.044**	**1.985 (1.406, 2.802)**	**<0.001**	**1.814 (1.268, 2.597)**	**0.001**
**PLT, ×109/L**	1.000 (0.997, 1.002)	0.709			0.999 (0.997, 1.001)	0.348		
**ALT, U/L**	1.001 (0.998, 1.004)	0.552			1.001 (0.999, 1.004)	0.326		
**TBIL, μmol/L**	0.988 (0.959, 1.017)	0.402			0.996 (0.973, 1.020)	0.760		
**ALB, g/L**	0.986 (0.944, 1.031)	0.547			1.005 (0.967, 1.044)	0.812		
**INR**	0.718 (0.085, 6.063)	0.761			0.603 (0.101, 3.590)	0.579		
**Child–Pugh classification, B**	**3.580 (1.133, 11.305)**	**0.030**	**4.522 (1.270, 16.107)**	**0.020**	2.024 (0.644, 6.363)	0.228		
**Approach of resection, anatomical**	1.283 (0.877, 1.878)	0.199			1.070 (0.775, 1.479)	0.681		
**Pringle maneuver, yes**	0.777 (0.450, 1.342)	0.366			0.869 (0.537, 1.405)	0.566		
**Blood loss, mL**	1.000 (1.000, 1.001)	0.717			1.000 (1.000, 1.000)	0.860		
**Blood transfusion, yes**	0.960 (0.237, 3.891)	0.955			0.976 (0.311, 3.062)	0.966		
**Operation time, min**	1.001 (0.999, 1.004)	0.296			1.001 (0.999, 1.003)	0.297		
**PA-TACE, yes**	**0.719 (0.491, 0.978)**	**0.032**	**0.726 (0.475, 0.986)**	**0.043**	**0.688 (0.500, 0.947)**	**0.022**	**0.691 (0.500, 0.955)**	**0.025**
**Differentiation, grades 3-4**	1.260 (0.850, 1.869)	0.249			1.336 (0.959, 1.860)	0.086		
**Microsatellites, yes**	**1.432 (0.828, 2.476)**	**0.199**	1.194 (0.671, 2.123)	0.546	**1.734 (1.101, 2.730)**	**0.017**	**1.388 (0.868, 2.222)**	**0.171**
**Cirrhosis, yes**	0.913 (0.625, 1.334)	0.638			1.088 (0.790, 1.498)	0.604		

RFS, recurrence free survival; OS, overall survival; HCC, hepatocellular carcinoma; PSM, propensity score matching; BMI, body mass index; HBsAg, hepatitis B surface antigen; AFP, alpha-fetoprotein; TBIL, total bilirubin; TP, total protein; ALB, albumin; ALT, alanine aminotransferase; PLT, platelets; INR, international normalized ratio; PA-TACE, postoperative adjuvant transhepatic arterial chemoembolization; MPVI, microportal vein invasion.

Bold values, statistical significant.

On the other hand, the RFS and OS of HCC patients with MPVI showed no significant differences between the PA-TACE and LR alone groups in both log-rank test (median RFS, 10.4 vs. 6.5 months, p=0.13; median OS, 27.3 vs. 17.9 months, p=0.26; **Figures 3B, D**) and regression analysis (RFS, 0.131; OS, p=0.266; **Table 5**). The correlated results of the crude cohort were reported in **Supplementary File**, **Figure S2B, D** and **Table S3**.

**Table 5:** Univariate analysis for RFS and OS of HCC patients with MPVI in the PSM cohort.

Variables	RFS		OS	
	HR (95% CI)	p value	HR (95% CI)	p value
**SEX, male**	0.690 (0.363, 1.310)	0.257	0.756 (0.375, 1.523)	0.434
**Age, y**	0.998 (0.978, 1.019)	0.877	1.004 (0.982, 1.026)	0.754
**BMI, kg/m2**	1.033 (0.962, 1.110)	0.368	1.015 (0.941, 1.095)	0.700
**Alcohol consumption, yes**	0.998 (0.642, 1.550)	0.993	1.189 (0.740, 1.912)	0.474
**Hypertension, yes**	1.054 (0.426, 2.610)	0.909	1.822 (0.784, 4.236)	0.163
**Diabetes, yes**	0.312 (0.043, 2.246)	0.248	0.957 (0.234, 3.917)	0.951
**HBsAg, positive**	1.587 (0.874, 2.881)	0.129	1.564 (0.818, 2.992)	0.176
**AFP, <400 ng/mL**	0.680 (0.438, 1.057)	0.087	0.725 (0.447, 1.174)	0.190
**Diameter, cm**	1.009 (0.942, 1.081)	0.799	1.050 (0.979, 1.127)	0.172
**Number of tumors, multiple**	1.295 (0.834, 2.012)	0.250	1.111 (0.683, 1.808)	0.671
**PLT, ×109/L**	1.000 (0.998, 1.003)	0.922	1.000 (0.997, 1.003)	0.933
**ALT, U/L**	0.996 (0.990, 1.003)	0.229	0.996 (0.989, 1.003)	0.254
**TBIL, μmol/L**	1.025 (1.005, 1.046)	0.013	**1.028 (1.004, 1.052)**	**0.020**
**ALB, g/L**	0.994 (0.944, 1.047)	0.822	0.992 (0.937, 1.050)	0.783
**INR**	1.315 (0.113, 15.273)	0.827	0.214 (0.013, 3.431)	0.276
**Child–Pugh classification, B**	0.547 (0.076, 3.961)	0.551	0.718 (0.099, 5.231)	0.744
**Approach of resection, anatomical**	1.255 (0.801, 1.965)	0.321	1.426 (0.886, 2.297)	0.144
**Pringle maneuver, yes**	0.767 (0.436, 1.352)	0.359	1.255 (0.653, 2.412)	0.496
**Blood loss, mL**	1.000 (1.000, 1.000)	0.920	1.000 (1.000, 1.000)	0.491
**Blood transfusion, yes**	1.315 (0.633, 2.733)	0.462	1.763 (0.843, 3.688)	0.132
**Operation time, min**	1.002 (0.999, 1.005)	0.172	1.000 (0.997, 1.003)	0.976
**PA-TACE, yes**	0.713 (0.460, 1.105)	0.131	0.763 (0.474, 1.229)	0.266
**Differentiation, grades 3-4**	0.813 (0.523, 1.266)	0.360	0.727 (0.451, 1.170)	0.189
**Microsatellites, yes**	1.691 (0.976, 2.930)	0.061	**2.279 (1.308, 3.971)**	**0.004**
**Cirrhosis, yes**	1.233 (0.800, 1.900)	0.342	1.217 (0.760, 1.949)	0.413

RFS, recurrence free survival; OS, overall survival; HCC, hepatocellular carcinoma; PSM, propensity score matching; BMI, body mass index; HBsAg, hepatitis B surface antigen; AFP, alpha-fetoprotein; TBIL, total bilirubin; TP, total protein; ALB, albumin; ALT, alanine aminotransferase; PLT, platelets; INR, international normalized ratio; PA-TACE, postoperative adjuvant transhepatic arterial chemoembolization; MPVI, microportal vein invasion.

Bold values, statistical significant.

**Figure 4:**
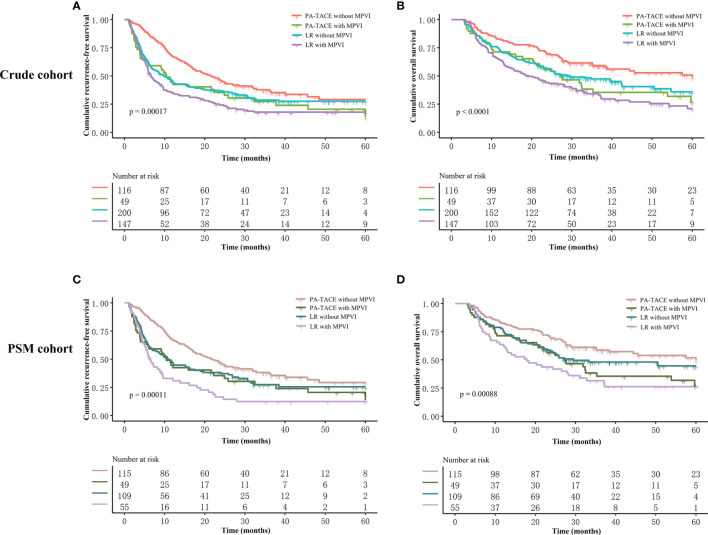
Kaplan–Meier analysis of RFS and OS stratified by all subgroups before and after PSM. **(A)** RFS stratified by treatment and MPVI presence in crude cohort. **(B)** OS stratified by treatment and MPVI presence in crude cohort. **(C)** RFS stratified by treatment and MPVI presence in PSM cohort. **(D)** OS stratified by treatment and MPVI presence in PSM cohort.

The Kaplan–Meier curves, RFS and OS stratified by treatment and the presence or absence of MPVI are displayed in **Figure 4** and **Table 6**. In a word, the RFS and OS remained better for the PA-TACE group than for the LR alone group in HCC patients without MPVI, which was not observed in HCC patients with the presence of MPVI.

**Table 6:** recurrence-free survival and overall survival of HCC patients stratified by treatment before and after PSM.

RFS in crude cohort					
Stratification		Median time	1-year rate	3-year rate	5-year rate
Overall	LR alone	8.2	41.1%	24.1%	23.5%
	PA-TACE	18.9	60.5%	34.8%	26.7%
Without MPVI	LR alone	9.8	45.4%	28.6%	27.5%
	PA-TACE	21.5	67.2%	37.8%	29.1%
With MPVI	LR alone	6.6	35.3%	17.9%	17.9%
	PA-TACE	10.4	44.6%	27.3%	13.7%
**OS in crude cohort**					
**Stratification**		**Median time**	**1-year rate**	**3-year rate**	**5-year rate**
Overall	LR alone	24.5	69.5%	41.7%	28.8%
	PA-TACE	44.4	79.4%	52.4%	42.3%
Without MPVI	LR alone	29.5	73.0%	47.4%	35.9%
	PA-TACE	58.0	82.8%	59.2%	48.6%
With MPVI	LR alone	19.1	64.6%	34.2%	21.0%
	PA-TACE	27.3	71.4%	35.3%	27.3%
**RFS in PSM cohort**					
**Stratification**		**Median time**	**1-year rate**	**3-year rate**	**5-year rate**
Overall	LR alone	8.7	42.10%	22.80%	21.60%
	PA-TACE	19.0	60.30%	35.00%	24.40%
Without MPVI	LR alone	10.9	46.50%	27.40%	25.50%
	PA-TACE	21.7	67.00%	38.10%	29.30%
With MPVI	LR alone	6.5	32.90%	12.40%	12.40%
	PA-TACE	10.4	44.60%	27.30%	13.70%
**OS in PSM cohort**					
**Stratification**		**Median time**	**1-year rate**	**3-year rate**	**5-year rate**
Overall	LR alone	25.6	71.30%	42.30%	38.00%
	PA-TACE	44.4	79.30%	52.10%	42.90%
Without MPVI	LR alone	29.5	75.20%	48.10%	44.60%
	PA-TACE	57.5	82.60%	58.80%	49.60%
With MPVI	LR alone	17.9	63.60%	31.50%	26.30%
	PA-TACE	27.3	71.40%	35.40%	27.30%

HCC, hepatocellular carcinoma; PSM, propensity score matching; MPVI, microportal vein invasion.

## Discussion

To the best of our knowledge, this study is the first to analyze the effect of PA-TACE on RFS and OS outcomes among HCC patients without or without MPVI. In the entire cohort, PA-TACE displayed consistent benefits in terms of improvements in RFS and OS outcomes both before and after PSM, which was subsequently confirmed in the subgroup analysis of HCC patients without MPVI. Intriguingly, this beneficial effect of PA-TACE in terms of improving RFS and OS was not observed in the subgroup of HCC patients with MPVI. The subclassification of patients based on MVI status (presence or absence) was also identified as an independent risk factor for HCC patients with MVI. These findings provide new information that can help identify suitable patient candidates for PA-TACE and guide clinical decisions.

MVI is a histopathological feature with an incidence of 15% to 74.4% (20, 21) and is routinely assessed in all patients who undergo liver resection. Despite the controversy regarding population selection, the predictive capability of MVI has been widely acknowledged and applied. Several studies have reported that MVI is significantly related to poor prognosis in patients with solitary HCC tumors ≤2 cm in size and stage II (based on TNM stage) disease (22–24). Recent studies have attempted to promote the application of MVI by further subclassification according to the number of tumor cells and infiltrated vessels, the distance of infiltrated vessels to the margin of the lesion, and the type of infiltrated vessels (25–29). For example, in the Practice Guidelines for the Pathological Diagnosis of Primary Liver Cancer developed by China (25), MVI is evaluated based on the number and distribution of invaded vessels as follows: 1) no MVI; 2) M1: <5 MVI and ≤1 cm away from tumor tissues; 3) M2: >5 MVI or >1 cm away from tumor tissues. However, the existing MVI classification uses parameters that are difficult to evaluate and generalize in routine clinical-pathological settings, which further complicates validation (30).

Discussion over the subtypes of MVI is not a new topic; in 2009, Shirabe et al. reported that the presence of MPVI is a poor prognostic factor in patients with HCC, and anatomical resection is recommended for these patients (30). Similar results were proposed by Fujita et al. in 2011 (29). Both studies subsequently classified patients with MPVI into high-risk (≥2 invaded vessels) and low-risk (single invaded vessels), and the high-risk group displayed a worse prognosis than the low-risk group. Nevertheless, the subclassification of MVI in terms of the type of invaded vessels has not garnered much attention among the countless studies on the predictive capability of MVI. A recent multi-institutional study evaluated the value of anatomical resection in HCC patients with MPVI for the first time (31). Anatomical resection did not lead to any added benefit for RFS and OS over nonanatomical resection in HCC patients who underwent curative LR and in all subgroup analyses, indicating that MPVI might be an unprecedented predictor for poor prognosis. Another study, by Kang et al., classified MVI into MI, MHVI and MPVI and reported that HCC patients with either MI or MPVI who underwent curative LR had poorer prognoses than those without MVI. The 5-year RFS rates were 75%, 45% and 25% in the no MVI, MI and MPVI groups, respectively, whereas the corresponding 5-year OS rates were 90%, 78%, and 55%, respectively (12). The potential for MPVI to be a strong predictor of HCC patient prognosis after curative hepatectomy warrants the further documentation of invaded vessels when MVI presents.

We conducted this study to clarify whether the high recurrence rate caused by MPVI can be reduced by PA-TACE. Unfortunately, PA-TACE did not lead to any significant improvement in the prognosis of HCC patients with MPVI. PA-TACE is used as a routine procedure to prevent recurrence in high-risk populations and has been reported to be significantly effective under various circumstances. Despite numerous retrospective studies presenting partially conflicting findings but overall benefit in terms of prognosis (18, 32), several randomized controlled studies have proven the efficacy of PA-TACE in selected patients (33–36), but concerns over the quality of the study design have been raised (17). Thus, the actual impact of PA-TACE on patient survival outcomes remains controversial, and an in-depth study is warranted to ensure that appropriate patient populations are selected for PA-TACE. To date, studies on HCC subpopulations that have huge tumors (≥10 cm) (37), exceed the Milan criteria (38), have multinodular tumors (39), have hepatic vein invasion (40) and have portal vein tumor thrombus (15) receive various degrees of benefit from PA-TACE. On the other hand, the negative finding reported in our study is a reminder to apply other therapies or combined modalities to mitigate the excessively high recurrence rate and improve OS outcomes in HCC patients with MPVI.

The MPVI-related poor prognosis might be explained as follows. The tumor tissue is fed mainly by the neovascular arterial vessels and drained through the peritumor portal vein (29). As a result, the spread of tumor cells *via* the portal vein has generally been recognized as the main mechanism for intrahepatic metastasis (41, 42). The tumor cells may disseminate to more distant and larger vessels through the portal venous system, which could hamper the curative effect of R0 hepatectomy with a general resection margin (1-2 cm). Wide and/or narrow surgical margins have been shown to be irrelevant to the prognosis of patients with MPVI (10). As a consequence, the increased burden of disseminated tumor cells may be the reason for the reduced benefit of PA-TACE.

Our study has some limitations. First, the retrospective design of this study inevitably introduced selection bias. Second, the study was conducted with a single-center cohort. The negative findings in HCC patients with MPVI should be interpreted with caution. The relatively low incidence of MPVI resulted in few HCC patients with MPVI receiving PA-TACE; that is, the efficacy of PA-TACE on HCC patients with MPVI was not sufficiently observed or evaluated. The findings in this study should not be used as justification for the complete refusal to perform PA-TACE for HCC patients with MPVI. Nevertheless, our study illustrates the need to document the subtypes of MVI. Further prospective studies are needed to explore the efficacy of PA-TACE in larger numbers of HCC patients with MPVI. A well-designed randomized controlled study is warranted to validate the impact of PA-TACE based on MVI subclassification.

## Conclusion

In conclusion, among HCC patients who underwent curative liver resection without MPVI, those who received PA-TACE had better RFS and OS outcomes than those who underwent LR alone. For patients with MPVI, PA-TACE did not significantly improve either RFS or OS.

## Data Availability Statement

The raw data supporting the conclusions of this article will be made available by the authors, without undue reservation.

## Ethics Statement

The studies involving human participants were reviewed and approved by the Ethics Committee of West China Hospital of Sichuan University. Written informed consent for participation was not required for this study in accordance with the national legislation and the institutional requirements.

## Author Contributions

YQ and WW participated in the research design. YQ participated in the writing of the paper. YQ, TW, and SS participated in the performance of the research. YQ and YY participated in the data analysis. All authors contributed to the article and approved the submitted version.

## Funding

This research was supported by the Science and Technology Program of Sichuan Science and Technology Department (Nos. 2019YFS0029, 2019YFS0529), the National Natural Science Foundation of China (Nos. 81770566, 82000599) and the New Medical Technology Foundation of West China Hospital of Sichuan University (No. XJS2016004). The corresponding author WW is the guarantor.

## Conflict of Interest

The authors declare that the research was conducted in the absence of any commercial or financial relationships that could be construed as a potential conflict of interest.

## Publisher’s Note

All claims expressed in this article are solely those of the authors and do not necessarily represent those of their affiliated organizations, or those of the publisher, the editors and the reviewers. Any product that may be evaluated in this article, or claim that may be made by its manufacturer, is not guaranteed or endorsed by the publisher.

## Abbreviations

HCC, Hepatocellular carcinoma; LR, liver resection; PA-TACE, postoperative adjuvant transcatheter arterial chemoembolization; MVI, microscopic vascular invasion; OS, overall survival; MPVI, microscopic portal vein invasion; MI, microvessel invasion; RFS, recurrence-free survival; PSM, propensity score matching.
