# Supplementary material for: Efficacy of Postoperative Adjuvant Transcatheter Arterial Chemoembolization in Hepatocellular Carcinoma Patients With Microscopic Portal Vein Invasion
Source: Front Oncol. 2022 Jun 20;12:831614. doi: 10.3389/fonc.2022.831614 (PMC9252591; doi:10.3389/fonc.2022.831614)
Supplement: Supplementary file 1 [file DataSheet_1.docx]

# Efficacy of postoperative adjuvant transcatheter arterial chemoembolization in hepatocellular carcinoma patients with microscopic portal vein invasion

**Running title: Efficacy of PA-TACE in MPVI**

Yiwen Qiu^1^, MD, Yi Yang^1^, MD, Tao Wang^1^, MD, Shu Shen^1^, MD, Wentao Wang^1#^, MD, PhD.

^1^ Department of Liver Surgery & Liver Transplantation Center, West China Hospital of Sichuan University, Chengdu, P. R. China

^#^ Corresponding author: Wentao Wang, E-mail: wwtdoctor02@163.com.

Address: Department of Liver Surgery, West China Hospital of Sichuan University, 37 Guoxue Road, Chengdu 610041, P.R. China

Phone: +86 18980601895

Fax: +86 028-85422469

# Supplementary files

1. **Overall effect of PA-TACE on the RFS and OS outcomes of HCC patients with MVI**

In the crude cohort, the median RFS time was 8.2 months for patients who underwent LR alone, which was significantly shorter than the 18.9 months for patients who received PA-TACE (p=0.0018, Figure S1A). The median OS time of the HCC patients with MVI was 24.5 months for the LR alone group and 44.4 months for the PA-TACE group. The OS was significantly better for the PA-TACE group than for the LR alone group (1-, 3-, and 5-year rates of 79.4%, 52.4%, 42.3% vs. 69.5%, 41.7%, 28.8%, p=0.0019) (Figure S1B).

| 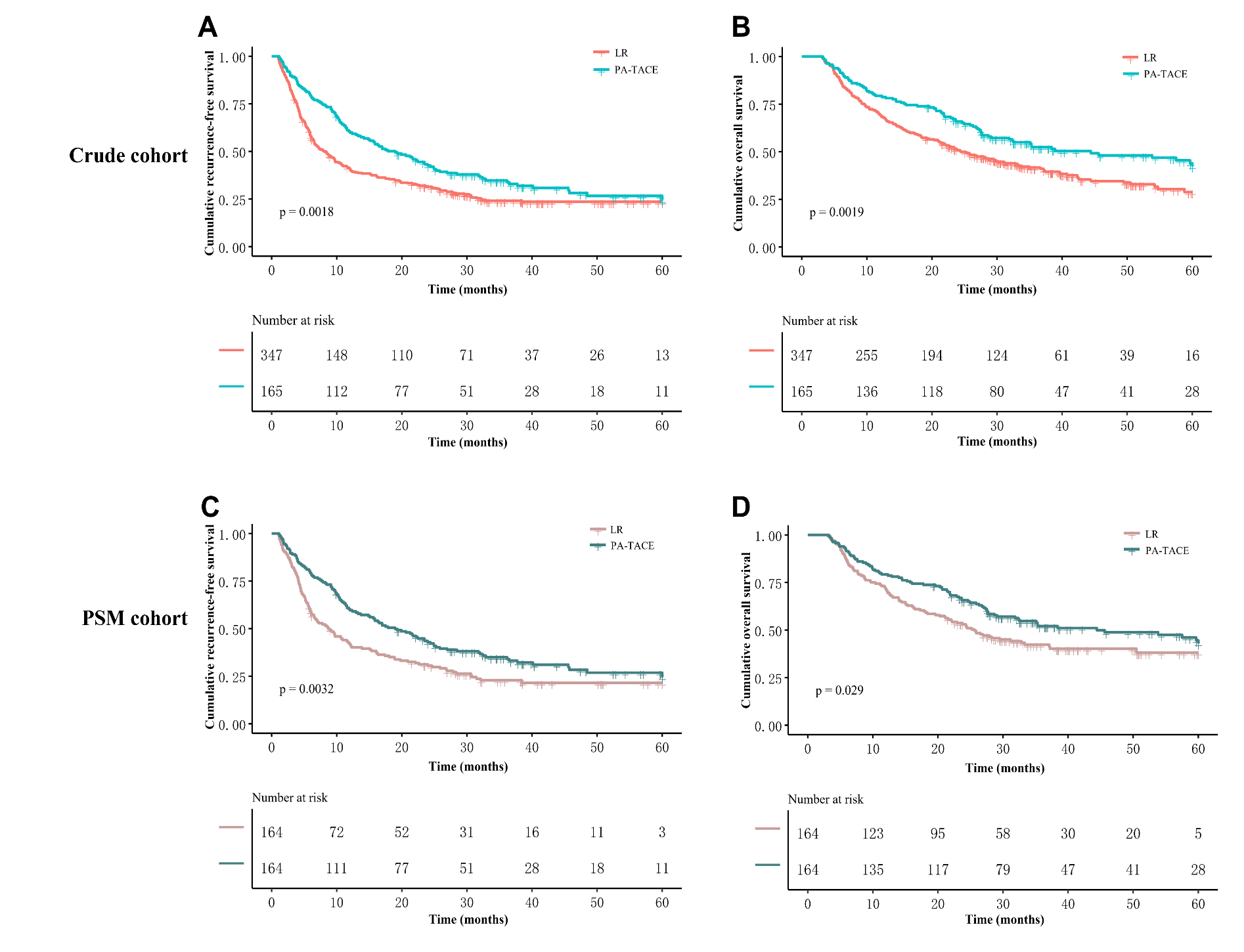 |
| --- |
| Figure S1. Kaplan–Meier analysis of RFS and OS stratified by treatment in the crude cohort. Patients who received PA-TACE had better RFS (A) and OS (B) than those who underwent liver resection alone. |

Univariate and multivariate regression analyses suggested that PA-TACE (HR 0.704, 95% CI 0.564-0.879, p= 0.002) and the presence of MPVI (HR 1.465, 95% CI 1.190-1.803, p<0.001) were independent predictors for RFS (Table S1). Regarding OS, PA-TACE (HR 0.734, 95% CI 0.567-0.951, p= 0.020) and the presence of MPVI (HR 1.435, 95% CI 1.136-1.812, p=0.011) were also identified as independent predictors.

**Table S1. Univariate and multivariate analysis for RFS and OS in HCC patients with MVI in the crude cohort.**

| **Variables** | **RFS** | | | | **OS** | | | |
| --- | --- | --- | --- | --- | --- | --- | --- | --- |
|  | **Univariate** | | **Multivariate** | | **Univariate** | | **Multivariate** | |
|  | **HR (95%CI)** | **p value** | **HR (95%CI)** | **p value** | **HR (95% CI)** | **p value** | **HR (95% CI)** | **p value** |
| **SEX, male** | 0.984 (0.726,1.332) | 0.915 |  |  | 1.287 (0.937, 1.768) | 0.119 |  |  |
| **Age, y** | 0.992 (0.983,1.001) | 0.087 |  |  | 0.995 (0.985, 1.005) | 0.362 |  |  |
| **BMI, kg/m^2^** | 1.008 (0.973,1.044) | 0.655 |  |  | 1.014 (0.975, 1.054) | 0.501 |  |  |
| **Alcohol consumption, yes** | 1.012 (0.822,1.246) | 0.912 |  |  | 1.005 (0.796, 1.269) | 0.966 |  |  |
| **Hypertension, yes** | 0.740 (0.525,1.045) | 0.087 |  |  | 0.666 (0.439, 1.012) | 0.057 |  |  |
| **Diabetes, yes** | 0.600 (0.351,1.023) | 0.061 |  |  | 0.709 (0.407, 1.237) | 0.226 |  |  |
| **HBsAg, positive** | **1.610 (1.194,2.171)** | **0.002** | **1.610 (1.194,2.171)** | **0.002** | **1.684 (1.193, 2.378)** | **0.003** | **1.639 (1.160, 2.316)** | **0.005** |
| **AFP, <400 ng/mL** | 0.823 (0.668,1.013) | 0.066 |  |  | 0.824 (0.653, 1.040) | 0.103 |  |  |
| **Diameter, cm** | 0.990 (0.955,1.026) | 0.582 |  |  | 0.991 (0.953, 1.031) | 0.663 |  |  |
| **Number of tumors, multiple** | **1.598 (1.290,1.979)** | **<0.001** | **1.598 (1.290,1.979)** | **<0.001** | **1.501 (1.183, 1.903)** | **<0.001** | 1.284 (0.993, 1.644) | 0.057 |
| **PLT, ×10^9^/L** | 1.000 (0.999,1.002) | 0.563 |  |  | 1.001 (0.999, 1.002) | 0.455 |  |  |
| **ALT, U/L** | 1.000 (0.998,1.002) | 0.871 |  |  | 1.000 (0.997, 1.002) | 0.849 |  |  |
| **TBIL, μmol/L** | 1.004 (0.999,1.010) | 0.129 |  |  | 1.002 (0.996, 1.008) | 0.525 |  |  |
| **ALB, g/L** | 0.995 (0.972,1.020) | 0.705 |  |  | 0.990 (0.964, 1.017) | 0.473 |  |  |
| **INR** | 1.065 (0.359,3.160) | 0.910 |  |  | 0.711 (0.211, 2.394) | 0.582 |  |  |
| **Child–Pugh classification, B** | 0.818 (0.338,1.980) | 0.655 |  |  | 1.115 (0.459, 2.705) | 0.810 |  |  |
| **Approach of resection, anatomical** | 1.032 (0.840,1.269) | 0.762 |  |  | 1.144 (0.909, 1.439) | 0.251 |  |  |
| **Pringle maneuver, yes** | 0.832 (0.622,1.113) | 0.215 |  |  | 0.854 (0.622, 1.174) | 0.332 |  |  |
| **Blood loss, mL** | 1.000 (1.000,1.000) | 0.400 |  |  | 1.000 (1.000, 1.000) | 0.164 |  |  |
| **Blood transfusion, yes** | 1.278 (0.894,1.828) | 0.179 |  |  | 1.357 (0.925, 1.992) | 0.118 |  |  |
| **Operation time, min** | **1.001 (1.000,1.003)** | **0.032** | **1.001 (1.000,1.003)** | **0.032** | 1.001 (1.000, 1.002) | 0.192 |  |  |
| **PA-TACE, yes** | **0.704 (0.564,0.879)** | **0.002** | **0.704 (0.564,0.879)** | **0.002** | **0.669 (0.519, 0.864)** | **0.002** | **0.734 (0.567, 0.951)** | **0.020** |
| **Differentiation, grades 3-4** | 1.064 (0.862,1.314) | 0.563 |  |  | 1.015 (0.802, 1.284) | 0.900 |  |  |
| **Microsatellites, yes** | **1.533 (1.179,1.993)** | **0.001** | **1.533 (1.179,1.993)** | **0.001** | **1.584 (1.192, 2.106)** | **0.002** | **1.394 (1.040, 1.869)** | **0.023** |
| **MPVI, yes** | **1.465 (1.190,1.803)** | **<0.001** | **1.465 (1.190,1.803)** | **<0.001** | **1.566 (1.245, 1.970** | **<0.001** | **1.435 (1.136, 1.812)** | **0.011** |
| **Cirrhosis, yes** | 1.121 (0.914,1.376) | 0.274 |  |  | 0.982 (0.781, 1.233) | 0.873 |  |  |

RFS: recurrence free survival; OS: overall survival; HCC: hepatocellular carcinoma; PSM: propensity score matching; BMI: body mass index; HBsAg: hepatitis B surface antigen; AFP: alpha-fetoprotein; TBIL: total bilirubin; TP: total protein; ALB: albumin; ALT: alanine aminotransferase; PLT: platelets; INR: international normalized ratio; PA-TACE: postoperative adjuvant transhepatic arterial chemoembolization; MPVI: microportal vein invasion.

1. **Effect of PA-TACE on RFS and OS outcomes based on the subclassification of MVI in the crude cohort.**

Among HCC patients without MPVI, PA-TACE resulted in a longer median RFS (21.5 vs. 9.8 months) and OS (44.4 vs. 21.5 months) time than LR alone. The OS was significantly better for the PA-TACE group than for the LR alone group (1-, 3-, 5-year rates of 67.2%, 37.8%, 29.1% vs. 45.4%, 28.6%, 27.5%, p=0.015) (Figure S2 AC). Univariate and multivariate regression analysis (Table S2) suggested that PA-TACE was a significant protective factor for both RFS (HR 0.716, 95% CI 0.539-0.952, p=0.021) and OS (HR 0.643, 95% CI 00.458-0.904, p=0.011).

On the other hand, among HCC patients with MPVI, the median RFS was 6.6 months for the LR alone group and 10.4 months for the PA-TACE group. The RFS was similar between the two groups (1-, 3-, and 5-year rates, 35.3%, 17.9%, and 17.9% vs. 44.6%, 27.3%, and 13.7%, p=0.34, Figure S2 B). OS showed similar results (median OS, 19.1 vs. 27.3; 1-, 3-, 5-year rates, 64.6%, 34.2%, 21.0% vs. 71.4%, 35.3%, 27.3%, p=0.27; Figure S2 D). Univariate and multivariate regression analysis (Table S3) suggested that PA-TACE had no significant effect on HCC patients with MPVI (Table S3).

| 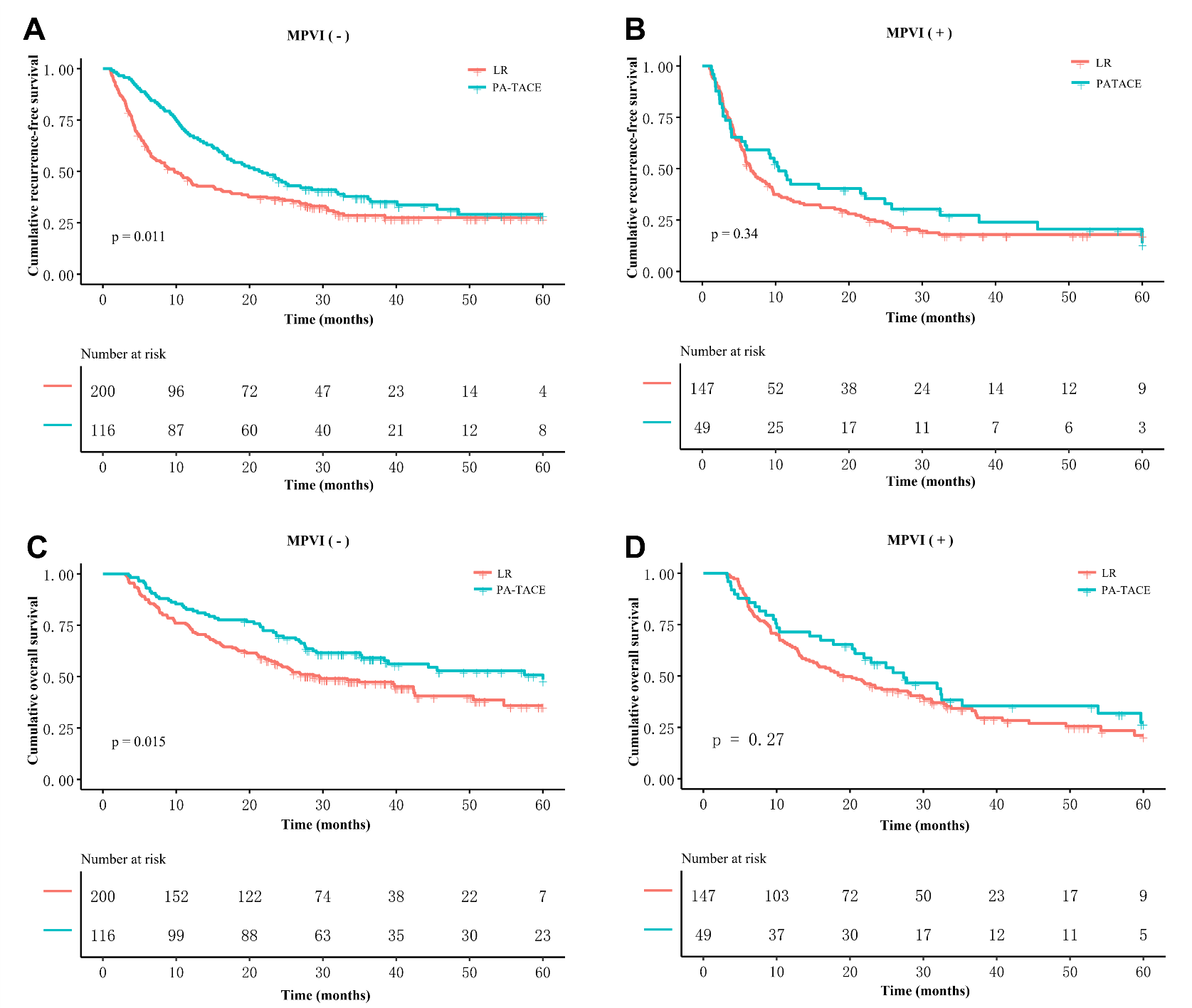 |
| --- |
| Figure S2. Kaplan–Meier analysis of RFS and OS stratified by treatment and the presence or absence of MPVI in the crude cohort. Patients without MPVI who received PA-TACE had better RFS (A) and OS (C) than those who underwent liver resection alone. The RFS (B) and OS (D) of patients with MPVI showed no significant differences between the PA-TACE and LR alone groups. |

**Table S2. Univariate and multivariate analysis for RFS and OS of HCC patients without MPVI in the crude cohort.**

| **Variables** | **RFS** | | | | **OS** | | | |
| --- | --- | --- | --- | --- | --- | --- | --- | --- |
|  | **Univariate** | | **Multivariate** | | **Univariate** | | **Multivariate** | |
|  | **HR (95% CI)** | **p value** | **HR (95% CI)** | **p value** | **HR (95% CI)** | **p value** | **HR (95% CI)** | **p value** |
| **SEX, male** | 0.942 (0.629, 1.411) | 0.773 |  |  | 1.400 (0.920, 2.131) | 0.116 |  |  |
| **Age, y** | 0.988 (0.976, 1.000) | 0.046 |  |  | **0.986 (0.972, 1.000)** | **0.046** | **0.982 (0.969, 0.996)** | **0.013** |
| **BMI, kg/m2** | 0.998 (0.952, 1.045) | 0.925 |  |  | 1.007 (0.954, 1.062) | 0.804 |  |  |
| **Alcohol consumption, yes** | 1.067 (0.812, 1.403) | 0.642 |  |  | 0.893 (0.648, 1.232) | 0.492 |  |  |
| **Hypertension, yes** | 0.758 (0.500, 1.151) | 0.194 |  |  | **0.538 (0.305, 0.949)** | **0.032** | 0.575 (0.326, 1.016) | 0.057 |
| **Diabetes, yes** | 0.663 (0.361, 1.218) | 0.185 |  |  | 0.706 (0.360, 1.384) | 0.310 |  |  |
| **HBsAg, positive** | **1.765 (1.186, 2.627)** | **0.005** | **1.839 (1.234, 2.741)** | **0.003** | **1.871 (1.159, 3.021)** | **0.010** | **1.926 (1.189, 3.117)** | **0.008** |
| **AFP, <400 ng/mL** | 0.823 (0.625, 1.083) | 0.163 |  |  | **0.795 (0.579, 1.092)** | **0.157** | 0.863 (0.626, 1.189) | 0.367 |
| **Diameter, cm** | 0.983 (0.934, 1.034) | 0.501 |  |  | 0.966 (0.912, 1.024) | 0.241 |  |  |
| **Number of tumors, multiple** | **1.595 (1.192, 2.135)** | **0.002** | **1.498 (1.111, 2.021)** | **0.008** | **1.524 (1.095, 2.123)** | **0.013** | **1.452 (1.038, 2.031)** | **0.030** |
| **PLT, ×109/L** | 0.999 (0.998, 1.001) | 0.532 |  |  | 1.000 (0.998, 1.002) | 0.949 |  |  |
| **ALT, U/L** | 1.000 (0.998, 1.003) | 0.794 |  |  | 1.000 (0.997, 1.003) | 0.977 |  |  |
| **TBIL, μmol/L** | 0.999 (0.980, 1.019) | 0.920 |  |  | 0.995 (0.972, 1.018) | 0.650 |  |  |
| **ALB, g/L** | 0.999 (0.968, 1.032) | 0.976 |  |  | 0.987 (0.951, 1.024) | 0.480 |  |  |
| **INR** | 1.051 (0.252, 4.377) | 0.946 |  |  | 1.175 (0.237, 5.836) | 0.844 |  |  |
| **Child–Pugh classification, B** | 2.547 (0.945, 6.862) | 0.064 |  |  | 3.839 (1.419, 10.384) | 0.008 |  |  |
| **Approach of resection, anatomical** | 1.060 (0.809, 1.390) | 0.672 |  |  | 1.230 (0.902, 1.677) | 0.191 |  |  |
| **Pringle maneuver, yes** | 0.864 (0.584, 1.278) | 0.464 |  |  | 0.758 (0.494, 1.161) | 0.203 |  |  |
| **Blood loss, mL** | 1.000 (1.000, 1.000) | 0.233 |  |  | 1.000 (1.000, 1.001) | 0.141 |  |  |
| **Blood transfusion, yes** | 1.359 (0.759, 2.433) | 0.302 |  |  | 1.038 (0.509, 2.115) | 0.918 |  |  |
| **Operation time, min** | 1.002 (1.000, 1.003) | 0.064 |  |  | 1.001 (1.000, 1.003) | 0.155 |  |  |
| **PA-TACE, yes** | **0.695 (0.525, 0.921)** | **0.011** | **0.716 (0.539, 0.952)** | **0.021** | **0.663 (0.475, 0.925)** | **0.015** | **0.643 (0.458, 0.904)** | **0.011** |
| **Differentiation, grades 3-4** | 1.177 (0.891, 1.556) | 0.250 |  |  | 1.135 (0.823, 1.564) | 0.440 |  |  |
| **Microsatellites, yes** | **1.467 (1.020, 2.109)** | **0.039** | **1.257 (0.866, 1.826)** | **0.229** | 1.292 (0.848, 1.968) | 0.233 |  |  |
| **Cirrhosis, yes** | 1.191 (0.909, 1.561) | 0.205 |  |  | 0.958 (0.703, 1.305) | 0.785 |  |  |

RFS: recurrence free survival; OS: overall survival; HCC: hepatocellular carcinoma; PSM: propensity score matching; BMI: body mass index; HBsAg: hepatitis B surface antigen; AFP: alpha-fetoprotein; TBIL: total bilirubin; TP: total protein; ALB: albumin; ALT: alanine aminotransferase; PLT: platelets; INR: international normalized ratio; PA-TACE: postoperative adjuvant transhepatic arterial chemoembolization; MPVI: microportal vein invasion.

**Table S3. Univariate analysis for RFS and OS of HCC patients with MPVI in the crude cohort.**

| **Variables** | **RFS** | | **OS** | |
| --- | --- | --- | --- | --- |
|  | **HR (95% CI)** | **p value** | **HR (95% CI)** | **p value** |
| **SEX, male** | 1.069 (0.674, 1.695) | 0.778 | 1.147 (0.706, 1.865) | 0.579 |
| **Age, y** | 0.998 (0.984, 1.012) | 0.758 | 1.006 (0.991, 1.021) | 0.457 |
| **BMI, kg/m2** | 1.033 (0.979, 1.089) | 0.235 | 1.033 (0.977, 1.093) | 0.254 |
| **Alcohol consumption, yes** | 0.915 (0.663, 1.263) | 0.590 | 1.116 (0.793, 1.570) | 0.530 |
| **Hypertension, yes** | 0.818 (0.443, 1.511) | 0.521 | 1.112 (0.600, 2.062) | 0.735 |
| **Diabetes, yes** | 0.539 (0.172, 1.692) | 0.289 | 1.019 (0.376, 2.761) | 0.970 |
| **HBsAg, positive** | 1.390 (0.883, 2.188) | 0.154 | 1.471 (0.893, 2.422) | 0.129 |
| **AFP, <400 ng/mL** | 0.785 (0.571, 1.081) | 0.138 | 0.832 (0.591, 1.173) | 0.294 |
| **Diameter, cm** | 0.979 (0.930, 1.031) | 0.420 | 1.000 (0.947, 1.056) | 0.995 |
| **Number of tumors, multiple** | **1.446 (1.050, 1.993)** | **0.024** | 1.304 (0.925, 1.840) | 0.130 |
| **PLT, ×109/L** | 1.001 (1.000, 1.003) | 0.144 | 1.001 (0.999, 1.003) | 0.302 |
| **ALT, U/L** | 0.999 (0.994, 1.003) | 0.532 | 1.000 (0.995, 1.004) | 0.852 |
| **TBIL, μmol/L** | 1.004 (0.998, 1.010) | 0.152 | 1.001 (0.996, 1.007) | 0.668 |
| **ALB, g/L** | 0.991 (0.956, 1.028) | 0.641 | 0.997 (0.958, 1.038) | 0.886 |
| **INR** | 1.070 (0.200, 5.733) | 0.937 | 0.344 (0.051, 2.294) | 0.270 |
| **Child–Pugh classification, B** | 0.184 (0.026, 1.322) | 0.093 | 0.217 (0.030, 1.561) | 0.129 |
| **Approach of resection, anatomical** | 0.992 (0.721, 1.366) | 0.962 | 1.034 (0.734, 1.456) | 0.848 |
| **Pringle maneuver, yes** | 0.822 (0.532, 1.272) | 0.380 | 1.061 (0.658, 1.711) | 0.808 |
| **Blood loss, mL** | 1.000 (1.000, 1.000) | 0.555 | 1.000 (1.000, 1.000) | 0.859 |
| **Blood transfusion, yes** | 1.047 (0.660, 1.659) | 0.846 | 1.297 (0.813, 2.069) | 0.275 |
| **Operation time, min** | 1.001 (0.999, 1.003) | 0.322 | 1.000 (0.998, 1.003) | 0.811 |
| **PA-TACE, yes** | 0.902 (0.652, 1.248) | 0.533 | 0.851 (0.602, 1.204) | 0.362 |
| **Differentiation, grades 3-4** | 0.837 (0.578, 1.212) | 0.345 | 0.796 (0.532, 1.191) | 0.268 |
| **Microsatellites, yes** | **1.512 (1.032, 2.217)** | **0.034** | **1.869 (1.262, 2.767)** | **0.002** |
| **Cirrhosis, yes** | 1.123 (0.818, 1.542) | 0.473 | 1.103 (0.785, 1.549) | 0.573 |

RFS: recurrence free survival; OS: overall survival; HCC: hepatocellular carcinoma; PSM: propensity score matching; BMI: body mass index; HBsAg: hepatitis B surface antigen; AFP: alpha-fetoprotein; TBIL: total bilirubin; TP: total protein; ALB: albumin; ALT: alanine aminotransferase; PLT: platelets; INR: international normalized ratio; PA-TACE: postoperative adjuvant transhepatic arterial chemoembolization; MPVI: microportal vein invasion.
